# Supplementary material for: Cardiometabolic Risk Factors and Physical Activity Patterns Maximizing Fitness and Minimizing Fatness Variation in Malaysian Adolescents: A Novel Application of Reduced Rank Regression
Source: Int J Environ Res Public Health. 2019 Nov 22;16(23):4662. doi: 10.3390/ijerph16234662 (PMC6926765; doi:10.3390/ijerph16234662)
Supplement: Supplementary file 1 [file ijerph-16-04662-s001.pdf]

Supplementary tables and figures

Information on Methods	
Table S1.docx	Timing of Variable Measures Across the Three Timepoints
Table S2.docx	Comparison of PA Patterns Across Sub-sets of the Total Sample at age 13y
Table S3.docx	Relation Between PA Pattern Score and Age in Boys and Girls
Table S4.docx	Variation in PA Pattern Score Across the Different Models in Boys and Girls
Table S5.docx	Cross-Sectional and Longitudinal Associations Between CRF Score and Cardiometabolic Health Outcomes in Boys
Table S6.docx	Cross-Sectional and Longitudinal Associations Between BMI and Cardiometabolic Health Outcomes in Boys
Table S7.docx	Cross-Sectional and Longitudinal Associations Between CRF Score and Cardiometabolic Health Outcomes in Girls
Table S8.docx	Cross-Sectional and Longitudinal Associations Between BMI and Cardiometabolic Health Outcomes in Girls
Table S9.docx	Cross-Sectional Associations Between PA Pattern Score and Cardiometabolic Health Outcomes in Boys, Malaysia.
Table S10.docx	Cross-Sectional Associations Between PA Pattern Score and Cardiometabolic Health Outcomes in Girls, Malaysia.
Figure S1.docx	Average trajectory of adolescents' PA from 13-17 years (N=1718)
Figure S2.docx	Flow chart of sample size recruited and used for analysis

Information on Methods

Linear change in PA pattern score over time was checked (Figure S1). To compute individual values of predicted baseline PA pattern score and PA pattern score change, for use in stage 2 models, individual-level random effects of the intercept (adolescent's deviation from the average PA pattern score at 13y) and the age coefficient (change in PA pattern score over time) were added to the average intercept and age coefficient respectively. The fit of the models was assessed with a Likelihood Ratio test comparing the deviance (-2*Log-Likelihood) of the models.

Regressions were conducted on unrestricted and complete case samples (see supplementary file for more information)., i.e. adolescents who had each health outcome measured at 13y and 17y. To examine whether associations between PA pattern score and cardiometabolic health may have been influenced by the varying sample sizes, regressions were repeated on a complete case sample of participants and on a complete case sample of adolescents who had data for all variables (all health outcomes across the three timepoints, PA pattern score/CRF score/BMI and all adjusted covariates, n_{boys}=162, n_{girls}=417).

Table S1: Timing of Variable Measures Across the Three Timepoints.

	2012 (13y)	2014 (15y)	2016 (17y)
**PA**	✓	✓	✓
**CRF**	✓	✓	✓
**BMI**	✓	✓	✓
**Cardiometabolic risk factors**			
**SBP**	✓	✓	✓
**DBP**	✓	✓	✓
**GLU**	✓	✓	✓
**TAG**	✓	✓	✓
**LDL**	✓	✓	✓
**WC**	✓	✓	✓
**BF**	✓	✓	✓
**Covariates**			
**Gender**	✓	✓	✓
**Ethnicity**	✓	✓	✓
**Place of residence**	✓		
**Smoking**	✓	✓	✓
**Asthma**	✓		
**Tanner Stage**	✓	✓	
**Sleep**	✓	✓	✓
**Screen time**	✓	✓	
**Parental education**	✓		
**Parental employment**	✓		
**Household income**	✓		

Abbreviations: BF, Body Fat; BMI, Body Mass Index; DBP, Diastolic Blood Pressure; GLU, Glucose; LDL, Low Density Lipoprotein; PA, Physical Activity; SBP, Systolic Blood Pressure; TAG, Triglycerides; WC, Waist Circumference.

Table S2: Comparison of PA Patterns Across Sub-sets of the Total Sample at age 13y.

13y		N	Explained variation (%)				Coefficient of congruence[4]	Correlation Coefficient[5]	
			Responses (total)	CRF score	BMI z-score[3]	PA predictor variables (total)		CRF score	BMI z-score[3]
**Total sample[1]**		1309	6.4	12.3	0.6	11.3	.	0.4**	0.04
**Gender**	Boys	497	6.7	6.6	6.8	6.2	0.82	0.3**	-0.3**
	Girls	812	3.3	6.4	0.2	6.7	0.76	0.4**	-0.1*
**Ethnicity**	Malay	1062	6.6	12.8	0.5	10.2	0.99	0.4**	-0.1*
	Other	249	11.4	16.2	6.6	15.1	0.89	0.4**	-0.2*
**Urbanicity**	Urban	702	6.9	12.6	1.2	11.6	0.94	0.4**	-0.1*
	Rural	607	8.8	16.4	1.2	10.2	0.97	0.4**	-0.1*
	Age (overall sample)[2]	3034	6.9	13.5	0.2	7.1	0.88	0.4**	-0.04*
**Age**	13y	1309	6.4	12.3	0.6	11.3	.	0.4**	0.04
	15y	834	9.8	18.9	0.6	10.5	0.91	0.4**	-0.1*
	17y	891	13.5	26.9	0.03	10.7	0.96	0.5**	0.02
**Random samples**	Random Sample 1	653	5.3	10.5	0.1	12.1	0.97	0.3**	-0.05
	Random Sample 2	656	9.1	17.4	0.8	9.7	0.98	0.4**	-0.1

Abbreviations: BMI, Body Mass Index; CRF, Cardiorespiratory Fitness.

[1]PA pattern derived from RRR with 23 predictor variables, i.e. types, location and timing of PA, and two intermediate variables, i.e. CRF and BMI.

[2]Includes all adolescents aged 13, 15 and 17y (n=1828), to compare the PA patterns across the individual age groups.

[3]BMI z-score was created by standardising absolute BMI for gender and age based on WHO growth reference data (22).

[4]Coefficient of congruence is calculated to compare the pattern loadings of a PA pattern derived from a population sub-set with the pattern loadings of the PA pattern derived from the total sample. A congruence coefficient of ≥0.95 indicates good similarity, 0.85-0.94 indicates fair similarity and <0.85 indicates no similarity.

[5]Pearson correlations adjusted for gender were performed between each intermediate variable and the PA pattern score. Correlations are weighed based on non-selection survey weights provided by MyHeARTs.

**P*<0.05, ***P*<0.001

Table S3: Relation Between PA Pattern Score and Age in Boys and Girls.

**Boys**							
	**n**	**PA pattern score (13y)[1]**	**95% CI**	***P***	**PA trajectory[2,3]**	**95% CI**	***P***
Model 1	675	-0.01	-0.113, 0.085	0.782			
Model 2	675	0.0	-0.099, 0.094	0.961	-0.01	-0.039, 0.024	0.624
Model 3	675	0.01	-0.120, 0.143	0.868	-0.01	-0.041, 0.024	0.624
Model 4	675	0.01	-0.115, 0.142	0.836	-0.01	-0.041, 0.025	0.628
**Girls**							
	**n**	**PA pattern score (13y)[4]**	**95% CI**	***P***	**PA Trajectory[2,3]**	**95% CI**	***P***
Model 1	1044	-0.18	-0.270,0.092	<0.001			
Model 2	1044	-0.05	-0.158,0.051	0.313	-0.08	-0.096, -0.057	<0.001
Model 3	1043	-0.08	-0.161,0.002	0.045	-0.08	-0.095, -0.056	<0.001
Model 4	1043	-0.08	-0.153,0.001	0.052	-0.08	-0.097, -0.057	<0.001

Abbreviations: CI, Confidence Interval; PA, Physical Activity.
[1]Predicted PA pattern score for Malay boys, living in urban areas at 13y. Boys' mean PA pattern score at 13y is 0.14±0.3.
[2]The difference in PA pattern score associated with 1-year change in age. Boys' average trajectory is -0.01±0.0.5 and girls' is -0.08±0.05.
[3]Age is centred at 13y
[4]Predicted PA pattern score for Malay girls, living in urban areas at 13y. Girls' mean PA pattern score at 13y is -0.07±0.32.
Model 1: 3-level random intercept, no slope
Model 2: 3-level random intercept, fixed slope for age
Model 3: 3-level random intercept, fixed slope for age - adjusted for ethnicity, urbanicity
Model 4: 3-level random intercept, random slope for age - adjusted for ethnicity, urbanicity

Table S4: Variation in PA Pattern Score Across the Different Models in Boys and Girls.

**BOYS**												
	**Model 1**			**Model 2**			**Model 3**			**Model 4**		
	**n=675**			**n=675**			**n=675**			**n=675**		
Derived from the models	**Estimate**	**95% CI**		**Estimate**	**95% CI**		**Estimate**	**95% CI**		**Estimate**	**95% CI**	
**Within adolescents' variance**	0.55	0.48	0.63	0.55	0.47	0.63	0.55	0.47	0.63	0.48	0.39	0.58
**Between school variance**	0.03	0.01	0.07	0.03	0.01	0.07	0.03	0.01	0.07	0.03	0.01	0.07
**Between adolescents' variance**	0.19	0.13	0.28	0.19	0.13	0.29	0.19	0.13	0.29	0.27	0.14	0.52
**Total variance**	0.77			0.77			0.77			0.77		
**Slope variance**	.	.	.	.	.	.	.	.	.	0.02	0	0.07
**Covariance (intercept, slope)**	.	.	.	.	.	.	.	.	.	-0.03	-0.09	0.03
**Intra-class correlation (Adolescent) (%)**	25.1			25.2			25.2			35.3		
**Deviance[1]**	6034			6033			6032			6025		
**GIRLS**												
	**Model 1**			**Model 2**			**Model 3**			**Model 4**		
	**n=1044**			**n=1044**			**n=1043**			**n=1043**		
Derived from the models	**Estimate**	**95% CI**		**Estimate**	**95% CI**		**Estimate**	**95% CI**		**Estimate**	**95% CI**	
**Within adolescents' variance**	0.52	0.45	0.61	0.49	0.42	0.57	0.49	0.42	0.57	0.46	0.4	0.54
**Between school variance**	0.03	0.01	0.06	0.02	0.01	0.05	0.02	0.01	0.05	0.02	0.01	0.05
**Between adolescents' variance**	0.12	0.09	0.17	0.14	0.1	0.19	0.14	0.1	0.19	0.26	0.21	0.33
**Total variance**	0.67			0.65			0.65			0.74		
**Slope variance**										0.01	0	0.01
**Covariance (intercept, slope)**										-0.04	-0.04	-0.03
**Intra-class correlation (Adolescent) (%)**	18.2			20.7			20.7			35.4		
**Deviance[1]**	11088			10947			10929			10861		

Abbreviations: CI, Confidence Interval.

Model 1: 3-level random intercept, no slope

Model 2: 3-level random intercept, fixed slope for age

Model 3: 3-level random intercept, fixed slope for age - adjusted for ethnicity, urbanicity

Model 4: 3-level random intercept, random slope for age - adjusted for ethnicity, urbanicity

[1]The fit of the models was assessed with a Likelihood Ratio test comparing the deviance (-2*Log-Likelihood) of the models.

Table S5: Cross-Sectional and Longitudinal Associations Between CRF Score and Cardiometabolic Health Outcomes in Boys.

	N	CRF 13y[1]	95% CI	*P*	N	CRF 15y[1]	95% CI	*P*	N	CRF 17y[1]	95% CI	*P*	N	CRF change[2,3]	95% CI	*P*
Health Outcomes (13y)																
**SBP (mmHg)**	417	-0.15	-0.24,-0.06	0.001												
**DBP (mmHg)**	417	-0.19	-0.26,-0.11	<0.001												
**GLU (mmol/L)**	417	-0.00	-0.01,0.00	0.11												
**LDL (mmol/L)**	417	-0.01	-0.02,-0.01	<0.001												
**TAG (mmol/L)**	417	-0.01	-0.02,-0.01	<0.001												
**WC (cm)**	417	-0.43	-0.54,-0.33	<0.001												
**BF (%)**	416	-0.50	-0.62,-0.37	<0.001												
Health Outcomes (15y)																
**SBP (mmHg)**	240	-0.17	-0.31,-0.02	0.028	227	-0.29	-0.39,-0.19	<0.001								
**DBP (mmHg)**	240	-0.12	-0.24,-0.01	0.035	227	-0.27	-0.36,-0.18	<0.001								
**GLU (mmol/L)**	245	-0.01	-0.02,0.00	0.162	225	-0.01	-0.00,-0.02	0.023								
**LDL (mmol/L)**	244	-0.01	-0.02,-0.00	0.02	224	-0.01	-0.00,-0.02	0.006								
**TAG (mmol/L)**	245	-0.02	-0.03,-0.00	0.009	225	-0.01	-0.01,-0.00	0.002								
**WC (cm)**	243	-0.45	-0.61,-0.29	<0.001	227	-0.38	-0.50,-0.27	<0.001								
**BF (%)**	243	-0.37	-0.49,-0.24	<0.001	227	-0.33	-0.43,-0.24	<0.001								
Health Outcomes (17y)																
**SBP (mmHg)**	180	-0.12	-0.30,0.06	0.175	220	-0.28	-0.44,-0.12	0.001	345	-0.23	0.32,-0.13	<0.001	165	-0.4	-0.9,0.1	0.108
**DBP (mmHg)**	180	-0.07	-0.17,0.03	0.191	220	-0.22	-0.36,-0.08	0.002	345	-0.16	-0.24,-0.07	<0.001	165	-0.3	-0.7,0.0	0.060
**GLU (mmol/L)**	178	-0.02	-0.05,0.01	0.241	218	-0.01	-0.03,0.00	0.14	343	-0.01	-0.01,0.00	0.07	164	-0.02	-0.03,-0.00	0.016
**LDL (mmol/L)**	177	-0.00	-0.02,0.00	0.297	217	-0.01	-0.01,0.00	0.134	342	-0.01	-0.01,-0.01	<0.001	163	-0.02	-0.04,-0.00	0.052
**TAG (mmol/L)**	178	-0.03	-0.07,0.01	0.196	218	-0.02	-0.04,0.00	0.102	343	-0.01	-0.02,0.00	0.07	164	-0.03	-0.05,-0.01	0.010
**WC (cm)**	180	-0.40	-0.59,-0.21	<0.001	220	-0.33	-0.56,-0.11	0.004	345	-0.27	-0.37,-0.18	<0.001	166	-0.3	-0.6,-0.05	0.040
**BF (%)**	180	-0.22	-0.33,-0.12	<0.001	220	-0.16	-0.29,-0.04	0.012	345	-0.13	-0.20,-0.06	<0.001	166	0.1	-0.5,0.7	0.621

Abbreviations: BF, Body Fat; CRF, Cardiorespiratory Fitness; CI, Confidence Interval; DBP, Diastolic Blood Pressure; GLU, Glucose; LDL, Low Density Lipoprotein; SBP, Systolic Blood Pressure; TAG, Triglycerides; WC, Waist Circumference.

All models are adjusted for ethnicity, urbanicity and dietary pattern score

[1]Cross-sectional associations: the difference in the health outcome associated with 1-unit increase in CRF score (in the corresponding year)

[2]Longitudinal associations: the difference in the cardiometabolic health outcome at 17y associated with 1-unit change in CRF score per year.

[3]Adjusted for ethnicity, urbanicity, dietary pattern score and cardiometabolic health outcome at 13y

Table S6: Cross-Sectional and Longitudinal Associations Between BMI and Cardiometabolic Health Outcomes in Boys.

	N	BMI 13y[1]	95% CI	*P*	N	BMI 15y[1]	95% CI	*P*	N	BMI 17y[1]	95% CI	*P*	N	BMI change[23]	95% CI	*P*
Health Outcomes (13y)																
**SBP (mmHg)**	484	0.84	0.62,1.06	<0.001												
**DBP (mmHg)**	484	0.64	0.47,0.81	<0.001												
**GLU (mmol/L)**	486	-0.01	-0.03,0.01	0.28												
**LDL (mmol/L)**	486	0.03	0.02,0.05	<0.001												
**TAG (mmol/L)**	486	0.04	0.03,0.05	<0.001												
**WC (cm)**	486	2.22	1.93,2.50	<0.001												
**BF (%)**	485	2.5	2.17,2.84	<0.001												
Health Outcome (15y)																
**SBP (mmHg)**	288	0.82	0.54,1.10	<0.001	413	0.91	0.70,1.13	<0.001								
**DBP (mmHg)**	288	0.57	0.33,0.81	<0.001	413	0.48	0.28,0.68	<0.001								
**GLU (mmol/L)**	296	0.0	-0.01,0.01	0.62	412	0.0	-0.01,0.01	0.95								
**LDL (mmol/L)**	295	0.03	0.02,0.05	<0.001	411	0.05	0.03,0.06	<0.001								
**TAG (mmol/L)**	296	0.03	0.02,0.05	<0.001	412	0.03	0.02,0.04	<0.001								
**WC (cm)**	290	2.26	1.90,2.63	<0.001	416	2.39	2.30,2.47	<0.001								
**BF (%)**	290	1.86	1.56,2.16	<0.001	416	2.04	1.94,2.14	<0.001								
Health Outcome (17y)																
**SBP (mmHg)**	198	0.96	0.69,1.24	<0.001	235	1.41	1.15,1.67	<0.001	225	1.38	1.13,1.63	<0.001	168	3.3	-1.4,8.0	0.2
**DBP (mmHg)**	198	0.49	0.25,0.73	<0.001	235	0.72	0.46,0.97	<0.001	225	0.69	0.43,0.95	<0.001	168	1.9	-1.1,5.0	0.2
**GLU (mmol/L)**	196	0.02	-0.01,0.04	0.19	233	0.01	-0.00,0.02	0.15	223	0.01	0.00,0.02	0.19	167	0	-0.1,0.1	0.9
**LDL (mmol/L)**	195	0.03	0.01,0.05	0.01	232	0.05	0.03,0.07	<0.001	222	0.05	0.03,0.07	<0.001	166	0.2	0.01,0.4	<0.001
**TAG (mmol/L)**	196	0.04	0.01,0.07	0.01	233	0.03	0.01,0.05	<0.001	223	0.03	0.02,0.05	<0.001	167	0.1	-0.0,0.2	0.1
**WC (cm)**	198	2.19	1.72,2.66	<0.001	235	2.51	2.35,2.68	<0.001	225	2.47	2.35,2.59	<0.001	169	5.4	1.0,9.8	<0.001
**BF (%)**	198	1.19	0.92,1.47	<0.001	235	1.38	1.23,1.53	<0.001	225	1.38	1.28,1.48	<0.001	169	4	0.4,7.7	<0.001

Abbreviations: BF, Body Fat; BMI, Body Mass Index; CI, Confidence Interval; DBP, Diastolic Blood Pressure; GLU, Glucose; LDL, Low Density Lipoprotein; SBP, Systolic Blood Pressure; TAG, Triglycerides; WC, Waist Circumference.

All models are adjusted for ethnicity, urbanicity and dietary pattern score

[1]Cross-sectional associations: the difference in the health outcome associated with 1-unit increase in BMI (in the corresponding year)

[2]Longitudinal associations: the difference in the cardiometabolic health outcome at 17y associated with 1-unit change in BMI per year.

[3]Adjusted for ethnicity, urbanicity, dietary pattern score and cardiometabolic health outcome at 13y

Table S7: Cross-Sectional and Longitudinal Associations Between CRF Score and Cardiometabolic Health Outcomes in Girls.

	N	CRF 13y[1]	95% CI	*P*	N	CRF 15y[1]	95% CI	*P*	N	CRF 17y[1]	95% CI	*P*	N	CRF change[23]	95% CI	*P*
Health Outcome (13y)																
**SBP (mmHg)**	638	-0.13	0.21,-0.04	0.003												
**DBP (mmHg)**	638	-0.05	-0.12,0.02	0.179												
**GLU (mmol/L)**	638	-0.0	-0.00,0.00	0.952												
**LDL (mmol/L)**	638	-0.0	-0.00,0.01	0.553												
**TAG (mmol/L)**	638	-0.01	-0.01,-0.00	0.001												
**WC (cm)**	637	-0.34	-0.42,-0.26	<0.001												
**BF (%)**	637	-0.32	-0.39,-0.25	<0.001												
Health Outcome (15y)																
**SBP (mmHg)**	448	-0.083	-0.17,0.01	0.088	681	-0.13	-0.19,-0.07	<0.001								
**DBP (mmHg)**	448	-0.081	-0.15,-0.01	0.022	681	-0.1	-0.15,-0.05	<0.001								
**GLU (mmol/L)**	451	-0.0	-0.00,0.00	0.861	680	-0.03	-0.39,0.33	0.87								
**LDL (mmol/L)**	451	-0.004	-0.00,0.00	0.12	680	-0.03	-0.39,0.33	0.867								
**TAG (mmol/L)**	451	-0.004	-0.00,-0.00	0.002	680	-0.03	-0.39,0.33	0.875								
**WC (cm)**	450	-0.273	-0.36,-0.18	<0.001	682	-0.22	-0.29,-0.16	<0.001								
**BF (%)**	450	-0.239	-0.30,-0.17	<0.001	682	-0.22	-0.27,-0.17	<0.001								
Health Outcome (17y)																
**SBP (mmHg)**	392	-0.121	-0.17,0.01	0.079	490	-0.168	-0.24,-0.09	<0.001	495	-0.2	-0.2,0.1	<0.001	414	-0.2	-0.3,0.03	0.471
**DBP (mmHg)**	392	-0.062	-0.14,0.02	0.135	490	-0.142	-0.19,-0.08	<0.001	495	-0.1	-0.2,-0.1	<0.001	414	-0.1	-0.3,0.03	0.636
**GLU (mmol/L)**	389	-0.002	-0.00,0.00	0.091	490	-0.006	-0.01,0.00	0.219	495	-0.0	-0.0,0.0	0.2	419	-0.01	-0.01,-0.00	0.043
**LDL (mmol/L)**	389	-0.003	-0.00,0.00	0.225	490	-0.006	-0.01,-0.00	0.002	495	-0.0	-0.0,-0.0	<0.001	419	-0.002	-0.006,0.01	0.867
**TAG (mmol/L)**	389	-0.006	-0.00,-0.00	0.001	490	-0.004	-0.00,-0.00	<0.001	495	-0.0	-0.0,-0.0	<0.001	419	-0.004	-0.009,0.001	0.417
**WC (cm)**	392	-0.27	-0.35,-0.19	<0.001	490	-0.226	-0.29,-0.15	<0.001	495	-0.2	-0.3,-0.2	<0.001	414	-0.2	-0.3,-0.2	<0.001
**BF (%)**	392	-0.227	-0.28,-0.17	<0.001	490	-0.164	-0.21,-0.11	<0.001	495	-0.2	-0.2,-0.1	<0.001	414	-0.1	-0.2,0.0	0.149

Abbreviations: BF, Body Fat; CRF, Cardiorespiratory Fitness; CI, Confidence Interval; DBP, Diastolic Blood Pressure; GLU, Glucose; LDL, Low Density Lipoprotein; SBP, Systolic Blood Pressure; TAG, Triglycerides; WC, Waist Circumference.

All models are adjusted for ethnicity, urbanicity and dietary pattern score

[1]Cross-sectional associations: the difference in the health outcome associated with 1-unit increase in CRF score (in the corresponding year)

[2]Longitudinal associations: the difference in the cardiometabolic health outcome at 17y associated with 1-unit change in CRF score per year.

[3]Adjusted for ethnicity, urbanicity, dietary pattern score and cardiometabolic health outcome at 13y

Table S8: Cross-Sectional and Longitudinal Associations Between BMI and Cardiometabolic Health Outcomes in Girls, Malaysia.

	N	BMI 13y[1]	95% CI	*P*	N	BMI 15y[1]	95% CI	*P*	N	BMI 17y[1]	95% CI	*P*	N	BMI change[23]	95% CI	*P*
Health Outcome (13y)																
**SBP (mmHg)**	756	0.94	0.77,1.11	<0.001												
**DBP (mmHg)**	756	0.72	0.57,0.88	<0.001												
**GLU (mmol/L)**	756	0.01	-0.01,0.02	0.33												
**LDL (mmol/L)**	756	0.0	-0.01,0.02	0.52												
**TAG (mmol/L)**	756	0.02	0.01,0.03	<0.001												
**WC (cm)**	756	2.07	1.97,2.16	<0.001												
**BF (%)**	756	2.19	2.10,2.27	<0.001												
Health Outcome (15y)																
**SBP (mmHg)**	535	0.81	0.56,1.06	<0.001	689	0.69	0.49,0.90	<0.001								
**DBP (mmHg)**	535	0.55	0.38,0.72	<0.001	689	0.47	0.33,0.62	<0.001								
**GLU (mmol/L)**	542	-0.0	-0.01,0.02	0.96	689	0.0	-0.01,0.02	0.49								
**LDL (mmol/L)**	542	0.01	-0.00,0.03	<0.001	689	0.02	0.01,0.03	<0.001								
**TAG (mmol/L)**	542	0.02	0.01,0.03	<0.001	689	0.03	0.02,0.03	<0.001								
**WC (cm)**	537	1.85	1.69,2.01	<0.001	691	1.95	1.85,2.05	<0.001								
**BF (%)**	537	1.73	1.63,1.83	<0.001	691	1.74	1.68,1.81	<0.001								
Health Outcome (17y)																
**SBP (mmHg)**	464	0.88	0.60,1.16	<0.001	496	0.9	0.6,1.1	<0.001	500	0.9	0.7,1.1	<0.001	419	3.8	2.0,5.6	<0.001
**DBP (mmHg)**	464	0.70	0.50,0.90	<0.001	496	0.7	0.5,0.8	<0.001	500	0.7	0.6,0.8	<0.001	419	2.7	1.2,4.3	<0.001
**GLU (mmol/L)**	461	0.02	0.00,0.04	0.02	496	0.0	0.0,0.0	0.07	500	0.0	0.0,0.0	0.05	424	0.1	0.0,0.2	<0.001
**LDL (mmol/L)**	461	0.03	0.01,0.04	<0.001	496	0.0	0.0,0.0	<0.001	500	0.0	0.0,0.0	<0.001	424	0.2	0.1,0.3	<0.001
**TAG (mmol/L)**	461	0.03	0.02,0.04	<0.001	496	0.0	0.0,0.0	<0.001	500	0.0	0.0,0.0	<0.001	424	0.2	0.1,0.3	<0.001
**WC (cm)**	464	1.94	1.75,2.12	<0.001	496	2.0	1.9,2.1	<0.001	500	1.9	1.8,2.0	<0.001	419	7.8	6.9,8.7	<0.001
**BF (%)**	464	1.53	1.41,1.65	<0.001	496	1.5	1.4,1.6	<0.001	500	1.5	1.4,1.5	<0.001	419	6.0	5.2,6.9	<0.001

Abbreviations: BF, Body Fat; BMI, Body Mass Index; CI, Confidence Interval; DBP, Diastolic Blood Pressure; GLU, Glucose; LDL, Low Density Lipoprotein; SBP, Systolic Blood Pressure; TAG, Triglycerides; WC, Waist Circumference.

All models are adjusted for ethnicity, urbanicity and dietary pattern score

[1]Cross-sectional associations: the difference in the health outcome associated with 1-unit increase in BMI (in the corresponding year)

[2]Longitudinal associations: the difference in the cardiometabolic health outcome at 17y associated with 1-unit change in BMI per year.

[3]Adjusted for ethnicity, urbanicity, dietary pattern score and cardiometabolic health outcome at 13y

Table S9: Cross-Sectional Associations Between PA Pattern Score and Cardiometabolic Health Outcomes in Boys, Malaysia.

	N	PA pattern score 2012	95% CIs	p-value	N	PA pattern score 2014	95% CIs	p-value	N	PA pattern score 2016	95% CIs	p-value
Health Outcome 2012												
**Systolic BP (mmHg)**	493	-0.183	-0.230,0.042	0.235								
**Diastolic BP (mmHg)**	493	-0.737	-1.649,0.224	0.657								
**Glucose (mmol/L)**	496	-0.06	-0.182,0.061	0.331								
**LDL (mmol/L)**	496	-0.023	-0.080,0.035	0.441								
**Triglycerides (mmol/L)**	496	-0.034	-0.064,-0.003	0.029								
**Waist circumference (cm)**	494	-1.096	-1.997,-0.006	0.04								
**%Body Fat**	493	-1.469	-2.484,-0.002	0.05								
Health Outcome 2014												
**Systolic BP (mmHg)**					286	-0.352	-1.523,0.820	0.555				
**Diastolic BP (mmHg)**					286	-0.69	-1.800,0.420	0.222				
**Glucose (mmol/L)**					290	0.009	-0.036,0.055	0.685				
**LDL (mmol/L)**					289	-0.044	-0.110,0.022	0.193				
**Triglycerides (mmol/L)**					290	-0.004	-0.047,0.039	0.845				
**Waist circumference (cm)**					288	-0.119	-1.214,0.977	0.831				
**%Body Fat**					288	-0.165	-1.155,0.825	0.743				
Health Outcome 2016												
**Systolic BP (mmHg)**									294	0.152	-1.542,1.846	0.86
**Diastolic BP (mmHg)**									294	-0.592	-2.063,0.880	0.429
**Glucose (mmol/L)**									292	0.044	-0.077,0.165	0.474
**LDL (mmol/L)**									291	-0.004	-0.078,0.071	0.925
**Triglycerides (mmol/L)**									292	0.061	-0.091,0.214	0.429
**Waist circumference (cm)**									294	0.835	-0.508,2.178	0.222
**%Body Fat**									294	-0.189	-1.149,0.770	0.698

Abbreviations: BF, Body Fat; BMI, Body Mass Index; CI, Confidence Interval; DBP, Diastolic Blood Pressure; GLU, Glucose; LDL, Low Density Lipoprotein; SBP, Systolic Blood Pressure; TAG, Triglycerides; WC, Waist Circumference.
All estimates come from regressions adjusted for ethnicity and urbanicity.

Table S10: Cross-Sectional Associations Between PA Pattern Score and Cardiometabolic Health Outcomes in Girls, Malaysia.

	N	PA pattern score 2012	95% CIs	p-value	N	PA pattern score 2014	95% CIs	p-value	N	PA pattern score 2016	95% CIs	p-value
Health Outcome 2012												
Systolic BP (mmHg)	804	-0.945	-2.063,0.173	0.097								
Diastolic BP (mmHg)	804	-0.07	-0.965,0.824	0.877								
Glucose (mmol/L)	812	-0.04	-0.086,0.006	0.092								
LDL (mmol/L)	812	0.025	-0.041,0.090	0.464								
Triglycerides (mmol/L)	812	-0.008	-0.046,0.029	0.665								
Waist circumference (cm)	803	-0.883	-1.765,-0.001	0.05								
%Body Fat	803	-0.682	-1.649,0.284	0.166								
Health Outcome 2014												
Systolic BP (mmHg)					539	-0.953	-2.478,0.572	0.22				
Diastolic BP (mmHg)					539	0.108	-1.113,1.329	0.862				
Glucose (mmol/L)					541	-0.011	-0.074,0.053	0.744				
LDL (mmol/L)					541	-0.07	-0.144,0.004	0.063				
Triglycerides (mmol/L)					541	0.017	-0.035,0.068	0.531				
Waist circumference (cm)					542	-0.652	-2.067,0.762	0.365				
%Body Fat					542	-0.56	-1.735,0.614	0.349				
Health Outcome 2016												
Systolic BP (mmHg)									596	0.962	-0.836,2.761	0.294
Diastolic BP (mmHg)									596	0.011	-1.400,1.423	0.987
Glucose (mmol/L)									594	0.048	-0.010,0.105	0.102
LDL (mmol/L)									594	0.012	-0.085,0.110	0.804
Triglycerides (mmol/L)									594	0.016	-0.036,0.068	0.552
Waist circumference (cm)									596	1.473	-0.023,2.969	0.054
%Body Fat									596	1.301	-0.068,2.534	0.05

Abbreviations: BF, Body Fat; BMI, Body Mass Index; CI, Confidence Interval; DBP, Diastolic Blood Pressure; GLU, Glucose; LDL, Low Density Lipoprotein; SBP, Systolic Blood Pressure; TAG, Triglycerides; WC, Waist Circumference.
All estimates come from regressions adjusted for ethnicity and urbanicity.

Figure S1: Average trajectory of adolescents' PA from 13-17 years (N=1718.

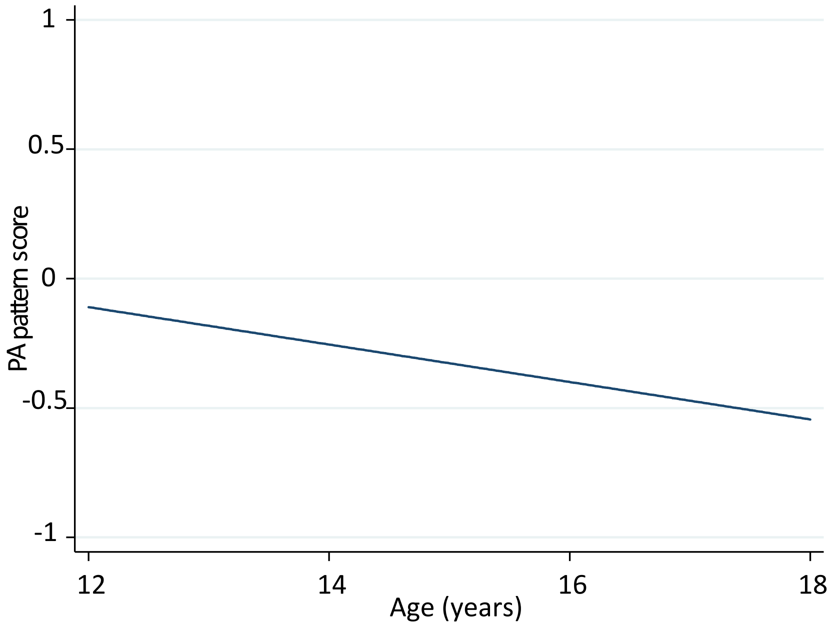

Figure S2: Flow chart of sample size recruited and used for analysis.

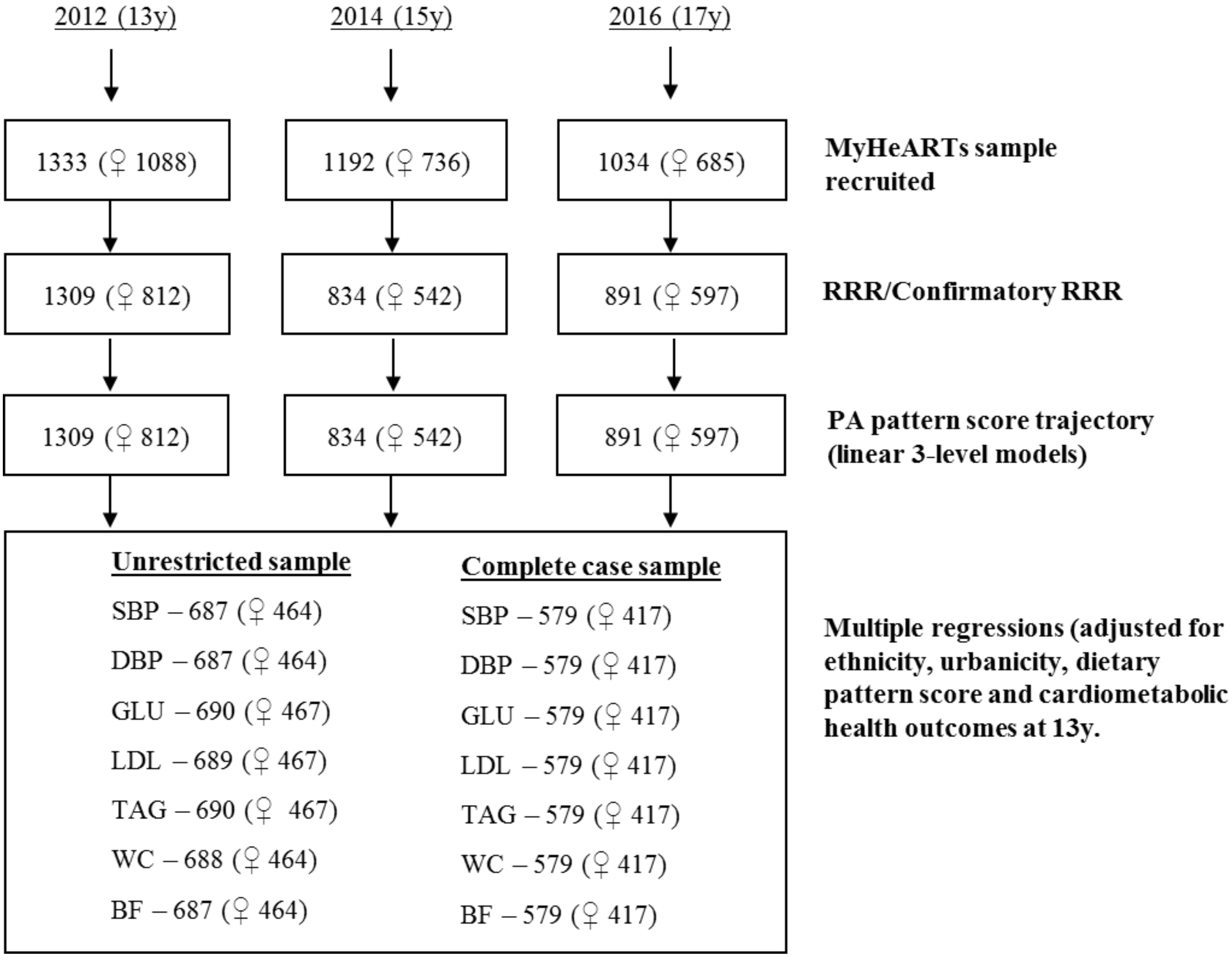

The unrestricted sample is made up of only those adolescents that had each risk factor measured at both 13y and 17y. The complete case sample is made up of adolescents who had data on all health outcomes across the three timepoints, PA pattern score/CRF score/BMI and all adjusted covariates. Abbreviations: BF – Body Fat, DBP – Diastolic blood pressure, GLU – Glucose, LDL – Low density lipoprotein, SBP – Systolic blood pressure, TAG – Triglycerides, WC – Waist circumference.
